# Organizational change readiness for team science: a pathway framework for team science success

**DOI:** 10.3389/fpsyg.2026.1761789

**Published:** 2026-03-06

**Authors:** Gaetano Romano Lotrecchiano, Deborah DiazGranados, Alfred Vitale, Margaret Browne-Huntt

**Affiliations:** 1 Department of Clinical Research and Leadership and of Pediatrics, George Washington University School of Medicine and Health Sciences, Washington, DC, United States; 2 Department of Psychiatry, School of Medicine, Virginia Commonwealth University, Richmond, VA, United States; 3 Program Evaluation & Continuous Improvement, Center for Clinical and Translational Science at Mayo Clinic, Rochester, MN, United States; 4 Department of Education Policy, Organization & Leadership, University of Illinois Urbana-Champaign, Champaign, IL, United States

**Keywords:** mentoring, organizations, readiness, sustainability, teams, training

## Abstract

Since the early 21st century, team science has significantly influenced emerging perspectives, processes, and expectations across various industries, human development, and scholarly activities. Research over the past 25 years—drawing on psychology, management, social psychology, anthropology, organizational sciences, and sociology—has shaped best practices and redefined how academics and practitioners approach collaborative work. Despite substantial progress in understanding team dynamics, a critical gap remains: the absence of a clearly defined professional pathway for team scientists and science workers. Specifically, there is a lack of a secure, structured pipeline that supports training, recognition, and career advancement in team science roles. This paper addresses the limited presence of systematic organizational strategies that help science workers from the early stages of their careers through long-term professional growth. As higher education confronts a shifting paradigm, institutions must now prepare students not only as disciplinary experts but also as collaborators equipped to thrive in a cross-disciplinary, problem-solving environment that embraces the workforce competencies employers seek. We examine the organizational requirements and challenges necessary to ensure success in science careers, emphasizing developmental training, robust mentorship, the professionalization and institutionalization of team structures, and sustaining collaboration. These elements are essential for preparing a workforce that is capable, resilient, and ready for the demands of future collaborative scientific endeavors.

## 1 Introduction

Team science represents a “re-embodiment of science” (Bradshaw and Bekoff, 2001), shifting the image of scientific progress away from the solitary genius toward collective intelligence. This paradigmatic transformation redefines what constitutes professional success in research: career trajectories increasingly dependent on an individual's capacity to collaborate effectively, integration across knowledge systems, and the contribution to meaningfully collective problem solving (Bennett and Gadlin, 2012; Foster, 2021; Klein et al., 2001; Stokols et al., 2008a). In this environment, career development must extend beyond cultivating technical or disciplinary expertise; it must instead foster scientists capable of bridging epistemic divides, communicating across institutional and cultural boundaries, and co-producing knowledge through dynamic, reciprocal engagement (Hall et al., 2018; Lotrecchiano, 2024; Lotrecchiano and Misra, 2018a). Building a sustainable career in team science thus requires recognition of diverse forms of contribution—from team facilitation and knowledge integration to conflict mediation and shared leadership—necessitating the rethinking of metrics used for career progression and evaluation (Cooke and Hilton, 2015; Falk-Krzesinski et al., 2011; Lotrecchiano et al., 2025).

Despite growing consensus that team science is essential to addressing complex societal and biomedical challenges (NASEM, 2025; Stipelman et al., 2010), organizational success in supporting such collaboration remains uneven. Many institutions launch interdisciplinary initiatives with ambitious goals but face systemic barriers: promotion systems that privilege individual accomplishments, rigid budget structures, weak project management capacity, and insufficient attention to psychological safety or inclusion can all undermine effectiveness (Bennett et al., 2018; Weiner, 2020; Weiner et al., 2020). Even when individual researchers are motivated and skilled, the absence of leadership commitment, incentive alignment, and sustained support often leads to inefficiency, mistrust, or premature project dissolution (Fiore, 2008; Lotrecchiano, 2016). This readiness gap illustrates that assembling the right expertise is necessary but insufficient; success depends equally on an organization's capacity to prepare its policies, culture, and infrastructure for collaboration (NASEM, 2025). Without deliberate readiness-building, the promise of team science remains only partially realized.

Organizational readiness, as defined in the team science literature, is a multidimensional, modifiable capacity rather than a static state. It reflects the extent to which an institution's people, infrastructure, culture, and incentives are aligned to form, sustain, and grow collaborative research teams (NASEM, 2025; Weiner et al., 2020). The National Academies report emphasizes that readiness operates across multiple levels—from individual motivation and team governance to institutional policies and system-level incentives such as funder expectations. Interventions that act on only one level, such as training individuals without reforming reward structures, are unlikely to produce durable change (Shea et al., 2014; Stokols et al., 2008b).

Among the strongest predictors of readiness is visible, strategic leadership commitment. Organizations that embed collaboration into strategic plans, promotion criteria, and resource allocation demonstrate institutional seriousness, while those treating collaboration as *ad hoc* or opportunistic rarely achieve sustained impact (Hall et al., 2018; NASEM, 2025). Closely related is the alignment of incentives and reward systems. Traditional evaluation criteria—individual authorship, principal investigator hierarchies, and siloed budgets—can inadvertently disincentivize teamwork (Falk-Krzesinski and Klein, 2017; Falk-Krzesinski et al., 2011). Redesigning these systems to formally recognize team-based contributions such as facilitation, data curation, and cross-disciplinary synthesis constitutes a core readiness intervention (Bennett et al., 2018; Cooke and Hilton, 2015).

Operational infrastructure is equally critical. Institutions that invest in shared administrative services, project management personnel, data-sharing platforms, and dedicated collaboration coordinators demonstrate higher readiness for interdisciplinary research (Fiore, 2008; NASEM, 2025). Workforce development further reinforces this foundation: training faculty, staff, and trainees in collaborative communication, conflict resolution, and team processes—and establishing legitimate career paths for team scientists—strengthens institutional capacity and continuity (Hall et al., 2018; Lotrecchiano and Misra, 2018b).

Cultural readiness forms the human substrate of effective team science. Psychological safety, equity, and trust are identified as non-negotiable preconditions for collaboration (Edmonston, 1999; NASEM, 2025). Diverse teams do not self-organize effectively unless organizations intentionally cultivate inclusive norms, shared power structures, and open communication. Mechanisms such as team charters, explicit governance models, and written agreements on authorship and conflict resolution function as structural safeguards that reduce ambiguity before it escalates into tension (Bennett and Gadlin, 2012; Bennett et al., 2014; Stipelman et al., 2010).

Finally, readiness is not a one-time achievement but an ongoing process of assessment and adaptation (Lotrecchiano et al., 2025). Institutions that regularly measure team functioning and readiness—using diagnostics such as the Organizational Readiness for Implementing Change (Shea et al., 2014) or other feedback loops—are better equipped to sustain progress. Conversely, organizations that rely solely on short-term, grant-dependent collaboration models remain structurally fragile (NASEM, 2025; Weiner et al., 2020).

In sum, organizational readiness for team science is best understood as an ecosystem condition: an interplay of leadership strategy, resources, culture, incentives, and infrastructure. It is both measurable and improvable through coordinated institutional action, rather than reliant on individual goodwill alone (Lotrecchiano, 2016; Lotrecchiano et al., 2025; NASEM, 2025; Stokols et al., 2008b).

This paper strives to expose some of the key areas that contribute to the problem described above by considering elements of organizational readiness that have the potential to change cultures and enhance readiness and also comment on key areas that are part of the scientific and scholarly lifecycle in organizations. We do this by proposing a framework that can be helpful for organizations to consider their own readiness to support and encourage team science. What we are not able to do is to provide a straightforward set of remedies because science organizations vary greatly in composition and mission. We are confident however, that the components of team science effort represented in the framework among science workers remains transferable and that definitions, functions, and infrastructure within organizations is shared.

### 1.1 Organizational change readiness

Organizational readiness for change (ORC) has evolved into a foundational construct in implementation science, shifting attention from individual attitudes toward change to collective, organizational-level capacity and motivation to implement innovation (Haque et al., 2016; Miake-Lye et al., 2020). Weiner's seminal theory (2020) positioned ORC as a shared psychological state characterized by both change commitment (the collective resolve to implement the change) and change efficacy (the shared belief in the organization's ability to execute the change). This dual focus is a critical departure from earlier approaches that equated readiness merely with structural capacity or resource availability. For Weiner, readiness is fundamentally relational and socially constructed—emerging through organizational sense making rather than merely predicted by material conditions.

To operationalize this theory, (Shea et al. 2014) developed and psychometrically validated the Organizational Readiness for Implementing Change (ORIC) measure, assessing change commitment and efficacy at the collective level. Their work empirically confirmed Weiner's argument that readiness is not simply about having resources but about organizational members' shared confidence and determination—a psychological rather than logistical construct. Importantly, ORIC demonstrated sensitivity to organizational context, underscoring that readiness is situational and task-specific rather than a static organizational trait. (Nordin 2012) expanded the conversation by empirically linking leadership behavior and organizational commitment to change readiness in higher education, offering early evidence that readiness is significantly mediated by leadership's ability to both communicate vision and bolster affective commitment among staff. Their colleague's work pushed beyond structural-functional determinants to emphasize leader-driven relational dynamics and emotional alignment—hinting at what later scholarship would articulate more directly: that readiness cannot be mandated; it must be socially produced through influence and meaning-making (Nordin, 2012).

Weiner et al. clarified the maturation of ORC scholarship, distinguishing between what is well-established (the distinction between motivation and capability), what is overassumed (that readiness is always change-specific rather than partially dispositional), and what remains under-theorized. They called for deeper attention to multilevel interactions, emphasizing that readiness is not just an organizational phenomenon but nested within systems, teams, and implementation environments—requiring a move beyond single-level analysis.

Most recently, the systematic review by (Caci et al. 2025) confirmed that ORC has become one of the most empirically tested predictors of implementation success in healthcare but also concluded that current readiness interventions overwhelmingly focus on assessment rather than activation. They argue that the field now faces a developmental imperative: moving from diagnosing readiness to engineering it (Caci et al., 2025). Their review also identifies a glaring gap—most models insufficiently account for temporal dynamics (how readiness fluctuates during implementation) and power asymmetries, which shape whose “determination” and “confidence” determine organizational action.

Across this evolution, a consistent conceptual through line emerges, organizational readiness is not a precondition you have—it is a collective mindset you generate and sustain (Lotrecchiano et al., 2025; Lotrecchiano, 2024), shaped by leadership, shared beliefs, affective commitment, and organizational-level sense making. The field is now shifting from measuring readiness to strategically cultivating it in context-sensitive, dynamic, and relationally intelligent ways.

### 1.2 Organizational readiness for team science success

Across the last decade, research in team science has increasingly reframed team readiness as a dynamic, interactional, and psychologically mediated condition—not a static precursor to implementation, but a state that is actively produced through cognition, motivation, and context. (Lotrecchiano et al. 2016) were early and influential in redefining readiness in this way. Their scoping review and domain analysis argued that collaboration readiness emerges from individual motivational drivers—such as curiosity, altruism, recognition-seeking—but is simultaneously constrained by perceived threat indicators, including fear of credit loss, epistemic marginalization, or goal misalignment. Rather than treating readiness as an organizational property, they framed it as a fluctuating motivational landscape negotiated through perceived benefits vs. risks of interdependence. This conceptualization laid critical groundwork for later studies that view readiness as emergent, relational, and psychologically situated.

(Groulx et al. 2025) extend this thinking at the team-level, arguing that readiness to change is not reducible to individual willingness but instead is collectively enacted through reflexivity (i.e., conscious examination of team assumptions), temporal dynamics of tenure, and the extent to which a forward-orienting vision is explicitly shared. Significantly, their findings show tenure can foster alignment but also heighten stagnation unless accompanied by active reflexive practices—a pattern that echoes (Lotrecchiano 2024) emphasis on how threats to autonomy, status, or identity can undermine readiness even in otherwise cohesive teams. Readiness itself as a cognitive-motivational state produced in the present through dialogic work, not inherited from past structure alone (Groulx et al., 2025).

Samost-Williams et al. expand the readiness frame outward, demonstrating in the clinical diagnostic context that shared mental models, a strong theme in (Fiore et al., 2001)—particularly between patients and clinicians—are co-constructed rather than assumed (Samost-Williams et al., 2025). They show that readiness for high-stakes team cognition requires fostering “epistemic symmetry”: a recognition that patients are not passive information sources but co-interpreters of diagnostic uncertainty. This finding resonates deeply with Lotrecchiano et al.'s (2025) argument that individual perceptions of psychological threat vs. motivational reward shape willingness to collaborate. In diagnostic care, readiness is not merely cognitive alignment—it is a willingness to relinquish epistemic dominance, which is itself a motivational and identity-risk negotiation.

Finally, Molldrem et al., in their study of the implementation of the TeamMAPPS intervention, empirically validate that interventions cannot assume readiness; they must generate it (Molldrem et al., 2025). Their qualitative findings show that uptake of team science interventions depends on whether they activate motivational drivers and reduce perceived threats through psychological safety, contextual resonance, and a sense that participation will yield competence and contribution—precisely the motivational calculus outlined conceptually by (Lotrecchiano et al. 2016) nearly a decade earlier and the basis for team competency building interventions (Lotrecchiano et al., 2023)

Taken together, these four studies converge on a clear trajectory: readiness is not a prerequisite state but a process of motivational, cognitive, and relational alignment—continuously negotiated. Interventions, reflexive practices, and shared meaning-making are not built on readiness—they are how readiness is produced. This signals a paradigm shift: team science must design for readiness formation, not simply screen for its prior existence (Lotrecchiano, 2024).

### 1.3 System science and conceptualizing organizational change

Systems science provides a powerful lens for understanding organizational readiness as an emergent property of complex, interconnected structures rather than a discrete, linear precursor to change. In systems terms, readiness arises from dynamic interactions among individuals, teams, institutions, and broader sociotechnical environments, all of which influence one another through feedback loops and adaptive processes (Meadows, 2008; Sweeney and Sterman, 2000). This perspective challenges static or checklist-based approaches to readiness by highlighting how conditions shift as new information, relationships, and external pressures affect the system. Systems science therefore encourages leaders to identify leverage points, anticipate unintended consequences, and design adaptive strategies that evolve alongside the system.

Team science scholarship further clarifies how readiness materializes in organizations that depend on collaboration, interdisciplinarity, and collective intelligence. Readiness for team science is not simply a matter of individual skill or motivation; it is shaped by shared goals, psychological safety, communication norms, and the degree to which organizations support integrative work (Bedwell et al., 2012; NASEM, 2025). Because scientific teams function as complex adaptive systems (CAS), their readiness fluctuates based on shifting team compositions, disciplinary cultures, resource flows, and institutional expectations (Cilliers, 2013; Hall et al., 2018; Lotrecchiano and Misra, 2018a). Systems science thus complements team science by showing how collaborative capacity emerges from multi-level interactions (Börner et al., 2010)—not from isolated efforts—making readiness inherently relational and context-dependent.

In this context, organizational readiness becomes inseparable from the system conditions that enable high-functioning collaborative science. Complex adaptive systems theory (CAS) suggests that teams are most effective when organizations cultivate flexible structures, decentralized decision-making, and mechanisms for rapid learning and sensemaking (Holland, 2002; Uhl-Bien and Arena, 2018). Such conditions allow teams to navigate uncertainty, integrate diverse viewpoints, and innovate across disciplinary boundaries—key features of successful team science. Moreover, readiness for team science depends on the organization's ability to bridge cultural divides, align incentives for collaboration, and support the co-creation of new knowledge, practices, and identities (NASEM, 2025; Stokols et al., 2008a).

Finally, systems-informed strategies for cultivating readiness emphasize the need for sustained investments in the social, structural, and cognitive infrastructures that support team science. This includes building shared vocabularies, creating platforms for data sharing, developing governance models that balance autonomy with coordination, and fostering cultures that reward learning, humility, and mutual accountability (Plsek and Wilson, 2001; Senge, 2006). Over time, these practices generate a resilient organizational ecosystem in which readiness is continuously renewed rather than episodically assessed. When organizations integrate systems science principles into the design and support of team science environments, they enhance not only their capacity to manage specific change initiatives but also their long-term ability to adapt, collaborate, and thrive in complex scientific landscapes.

### 1.4 Forging a new way forward for team science through organizational readiness

A deep understanding of “multi-level systems” (Börner et al., 2010) is foundational for navigating and shaping a team science career. This perspective teaches professionals to see their individual roles within broader institutional and societal contexts, understanding how their work impacts and is impacted by various scales of collaboration. For career development, this means training scientists not just in their specific disciplinary tradition, but also in the dynamics of team formation, project management in complex settings, and the policy implications of interdisciplinary work. Furthermore, the inherent fluidity of team science projects demands significant “adaptability” (Calarco and Gurvis, 2011). Career development programs must therefore prioritize fostering mental agility, problem-solving skills, and a willingness to embrace iterative processes and shifting objectives. Without the capacity for effective self-regulation and strategic adjustment, a science worker's professional growth within dynamic team environments could be severely hampered (Ajzen, 1991).

Developing leadership and facilitation skills is another cornerstone of career advancement in team science. (Christie and Lingard 2001) highlight the need for leaders who can orchestrate diverse expertise toward a common goal. This translates directly to the professional development of team science workers, who must learn to lead not just through authority, but through influence, empathy, and effective communication. The ability to utilize complexity and network concepts to inform knowledge translation (Kitson et al., 2017) further underscores the necessity for career-focused training in understanding team dynamics, stakeholder engagement, and the intricate pathways through which collaborative knowledge is generated and applied. These are not merely soft skills, but critical competencies that differentiate successful team science careerists (Christie and Lingard, 2001; Kitson et al., 2017).

Institutional support for career development in team science is paramount. Ahn-Sook points to the importance of creating environments where collective learning about collaboration is actively encouraged (Ahn-Sook, 2000). For individual careers to flourish, institutions must provide formal and informal opportunities for shared reflection, feedback, and skill acquisition related to team dynamics. This is a social learning approach to organizational behavior (Davis and Luthans, 1980; Lotrecchiano et al., 2025; Schwandt, 2005), where individuals learn through observing, imitating, and interacting within their professional community. Career development in this light becomes a shared responsibility, with institutions offering mentoring programs, interdisciplinary workshops, and communities of practice where scientists can continually refine their collaborative competencies.

Finally, the widespread adoption and successful integration of team science into academic career pathways depend on what Greenhalgh et al., 2004 coined diffusion of innovations. For team science to be a viable and attractive career option, the innovative practices and collaborative achievements must be systematically recognized and rewarded by institutions (Greenhalgh et al., 2004). This means, in some sectors, advocating for and implementing revised promotion, tenure and advancement guidelines that explicitly value team publications, collaborative grant acquisition, mentorship within interdisciplinary teams, and contributions to shared data and infrastructure (Klein and Falk-Krzesinski, 2017). Without such systemic changes, individual career aspirations in team science may remain stifled by traditional, individualistic evaluation models. The successful diffusion of team science as a legitimate and highly valued career path requires clear communication of its benefits, sustained advocacy, and robust institutional policies that champion its unique contributions.

Charting a successful career path in team science demands a proactive and multifaceted approach. It requires individuals to cultivate adaptability, systems thinking, collaborative leadership, and strong communication skills (Brasier et al., 2023; Lotrecchiano et al., 2025; Lotrecchiano and Misra, 2018b; Mendell et al., 2024). Simultaneously, it necessitates that institutions evolve their career development frameworks to foster systems-level learning, provide comprehensive training in interdisciplinary collaboration, and formally recognize the unique and vital contributions of team science workers. By consciously nurturing both the individual and systemic elements, we can ensure that the next generation of researchers is well-equipped to drive scientific progress through the powerful and essential lens of team science.

## 2 A pathway framework

**Figure 1:**
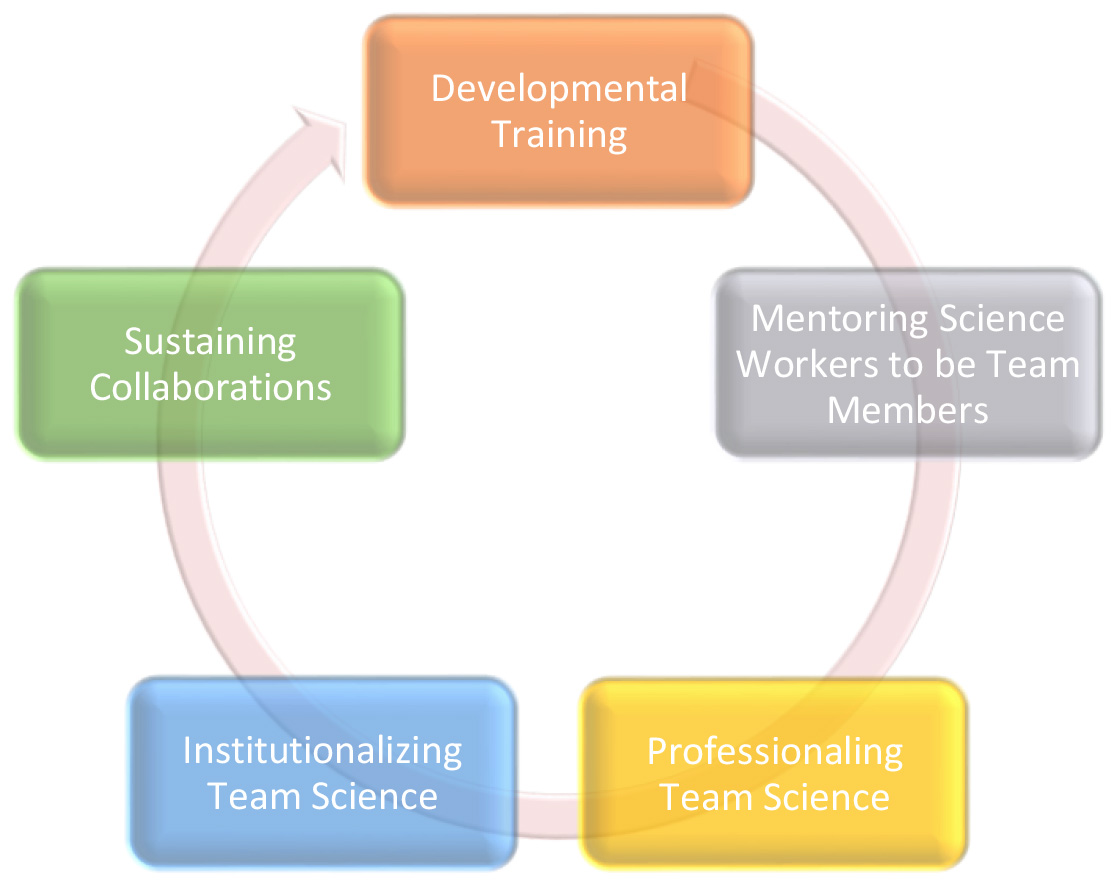
A pathway framework for team science success.

In keeping with the principles of organizational change, readiness, and systems science, this proposal outlines a framework that—if taken seriously—can support a holistic organizational redesign that strengthens individual career development, enhances team capacity, and promotes long-term sustainability. The five domains identified serve as areas which ensure that organizational change is the dual responsibility of individuals and the collective–organizational leaders and policy making and actors that exercise improved and intentional contributions (Burnes and Jackson, 2011). Through developmental training, mentoring science workers to become effective team members, professionalizing, and institutionalizing teams, and sustaining collaborative structures, this approach aims to cultivate a robust and enduring culture of team-based science that depends on the awareness of individual actor's roles and the organization requires necessary to affect change (Figure 1).

## 3 Developmental training: the training of new team science workers

Scientific research across all the sciences shares core epistemological assumptions whose practical embodiments are contextually bound within their particular domains. For team science, domain specificity intersects with team specificity—it is the makeup and dynamics of teams that must be navigated to facilitate scientific output. As noted in the NASEM 2025 report, team structures, research space (virtual, in-person, hybrid), research goals, team size and relative experience of its members, as well as the scope of research can vary greatly. Moreover, team productivity occurs differently in different stages (NASEM, 2025). Compounding this are the rapid changes to scientific technologies and the speed of scientific discoveries, which create ever-shifting goalposts for the preparation of effective scientific teaming. There is also the question of quantity—how much training is needed? And how much of that training needs to be generalized, vs. how much may require more of an immediately contextual specificity? And is it possible—or even desirable in some contexts—for more transactional skill attainment to have as much (or more) value than learning and internalizing team skills? These are interesting question, because some key team skills may only require a small amount of practice or exposure, such as those offered in workshops or short courses (Gilbert et al., 2017), mitigated by the learner's own learning tendencies and preferences. However, despite these contextual questions, there are general, fundamental competencies that cut across frameworks for team science development. Team science competencies, such as those developed for translational research teams (Lotrecchiano et al., 2020) have become more critical for scientific research but lack a cohesive set of validated standards for assessing these competencies and understanding how those are implemented in specific contexts will require more research (NASEM, 2025). Thus, the developmental training of team science workers in practice remains an evolving endeavor. Even so, there is a clear need for fundamental training that focuses on team science competency development. Overall, effective skills training needs relevance to the learner, demonstration of those skills, opportunities to practice those skills, and reflective feedback (Lotrecchiano et al., 2023; Salas and Cannon-Bowers, 2001). Implementing such an approach to training scientific teams may be possible in both academic and industry settings, though the relative cost-effectiveness and organizational investment and infrastructure will vary. Taking this into consideration, in this section, we will consider three important formative questions regarding the developmental training of new scientists and science workers: at what stage in science education should team science be introduced? Where should team science training be facilitated? And who should be leading team science training?

### 3.1 When should team science be introduced into science curricula?

The consideration of science as a team-endeavor may sometimes be implied or even documented in the historical literature, but at this very early stage of science education, collaboration on scientific projects among students is often more about classroom management and social development than about valorizing scientific teaming. Even with experiential learning opportunities, however, the focus on the science content knowledge required by standardized testing remains the highest priority—scientific collaboration or scientific teaming are certainly not part of the Next Generation Science Standards (National Research Council, 2012).

Beyond the high school classroom, competition to get into college reproduces the prioritization of the individual learner's achievements. Following admission, the course of study for most undergraduate STEM majors establishes narrow pathways for scientific career development heavy on content knowledge and, more commonly now, securing one of the limited opportunities to become involved in research and obtain coveted letters of recommendation for graduate programs. STEM majors are particularly vulnerable to setbacks, personal or academic, that can have adverse effects on their trajectories (Hedge, 2024) noted that 60% of students who have declared a STEM major will switch to non-STEM tracks or leave school altogether. Thus, the retention of STEM majors is a critical challenge to IHEs. As Cromley et al. explain, many factors influence under-achievement and dropping out of STEM courses or leaving science majors (Cromley et al., 2016). Additionally, among students leaving STEM majors, there is over-representation by women, as well as African American and Hispanic students, contributing to their later under-representation in scientific fields (National Student Clearinghouse Research Center, 2025)—which may encourage the institutionalization of skills training that could facilitate effective scientific teaming as students' progress into graduate programs and professional roles. Research on teamwork experiences in collaborative undergraduate research programs, which place students together as scientific teams pursuing research projects, have shown that such experiences improve retention and achievement in STEM fields (Russell et al., 2017) and emphasize the value of diverse perspectives and experiences (Agrawal et al., 2024). Yet for most, opportunities for collaboration are presented as part of hands-on learning—lab teams, project teams, and a variety of team activities that will certainly take place in any science program, with or without any structured team science training (usually without). Formal scientific training to prepare for the scientific workforce becomes a singular academic pursuit.

With developmentally formative years consuming contradictory and disproportionate messaging that science is mostly an individual but also, less importantly, a team activity must be reconciled in some way for the novice scientist to be able to engage in the scientific productivity expected at that stage. This broadly applies to most traditional scientific domains—the early accumulation of an enormous amount of fundamental content assessed uncompromisingly to adhere to scientific standards, followed by the attrition of potential scientists through rigorous and stressful undergraduate science courses, leading to the “cream rising to the top” and gaining access to research opportunities in graduate programs in their chosen field, with collaboration being half-heartedly praised for its ability to elevate scientific productivity and innovation while noted as being necessary (i.e., a requirement) for successful funding and careers in the academy or industry. Developing the so-called “T-shaped” scientist—a central goal in the field of translational science—is one attempt at reconciling the need for depth and breadth, which emphasis on systems thinking and boundary-crossing as well as disciplinary rigor (Wasko et al., 2024). The specific training of new scientists within other fields may temper the process of integrating individual and team contributions to align with the epistemology of their strand of scientific understanding. While training for new scientists varies across disciplines, we should also consider where structured team science training can and should take place.

### 3.2 What learning environment(s) facilitate optimal team science training?

As in all forms of training, learning occurs within a complex dynamic of contextual factors, and can be the result of deliberate efforts, unanticipated discoveries, or unconscious internalization through social, mentored, or individual engagement. In academic contexts, pedagogical strategies create parameters for the learning goals in a particular environment and direct attention toward relevant content or practice. But regardless of the open-endedness or willingness for an instructor to allow for organically derived forays into less relevant material, academia remains rooted to standards and grading and ultimately the prescribed curriculum sets the constraints. Conversely, outside of academia—where there is a myriad of learning opportunities—other incentives or directives constrain expectations for learning. In the case of team science training, where an end goal is to foster successful scientific teaming that exists primarily outside of classroom settings, there is a formative question we need to ask: should team science training take place in the classroom or should it take place in other learning environments? Because other people also have instrumentality in creating our developmental contexts, learning environments here also include proximal relationships—such as those of a mentor/mentee dyad, or across friends or peers—as well as sociocultural, institutional, or professional environments. The conditions for facilitating team science—including boundary-crossing and the integration of diverse viewpoints—are also critical mechanisms for the training and development of team scientists.

To some extent, however, the question about training environment has already been answered, and whether intentionally or unintentionally remains in plain view—because most “training” in team science takes place experientially in real-time in the context of actual team settings. That is how many people would answer the question of “how were you trained in team science”—offering some variation of “*i* was thrown into the pool and had to learn to swim or drown.” But, as we explore in this paper, developing team scientists is a fluid, contextually driven and idiosyncratic enterprise nestled within ever-evolving organizational ecosystems constituted by social, institutional, and pedagogical constraints. Immersion in scientific teams will be largely unavoidable. But institutionalizing a team-science conducive environment, an attainable goal for organizations, can mitigate some of the challenges faced by scientific teams. As noted earlier, organizational ecosystems are optimally conducive to team science when they can be flexible and responsive and create opportunities to foster and incentivize collaboration through supportive cognitive, social, and disciplinary infrastructures.

### 3.3 Who should train team scientists?

In the absence of academic credentials establishing some form of validated field of rank for instructors, or set of consistent and accepted academic standards for training team science, a third question arises: who is qualified to be a trainer? If training in team science is organized in interdisciplinary academic settings, this brings some degree of institutional challenges as it calls for cross-division (e.g., med school and business school collaboration) coordination that may cross policy or funding differences. But as we have seen, the systemic integration of team science principles into institutional infrastructure, and the prioritizing cultures of collaboration may attenuate operational or administrative challenges.

Still, even in adaptive and flexible institutional environments that prioritize and incentivize team science, the training and development of team scientists relies on trainers and instructors, as well as mentors (explored later in this paper). Without formal credentialing, the qualifications for teaching team science are likely to be locally determined and constrained by available academic resources. How would a program identify a “good” team science instructor? Does the experience of a trainer bring more value to the training than the training of the trainer—in other words, do experienced team scientists who may not have formal team science training better prepare future team scientists than less practically experienced but well-trained or well-researched trainers? Of course, this represents a continuum of possible training epistemologies. The reality may be more fluid, though there has not yet been a comprehensive inventory of the relative training or experience of those who are responsible for training scientists to work within teams. The focus of most research on team science training is on pedagogical strategies, implementation, and evaluation, or on quantifying appropriate metrics or indicators of team skills. In a 2001 review of a decade of literature on the science of training by Salas and Cannon-Bowers, several fundamental, beneficial trainer qualities are suggested. These include the ability to incorporate an evidence-based training design, with a reflective cycle to allow for iterative instructional strategies, as well as expertise in leading simulation or event-based opportunities for learning, grounding in some educational theory, understanding of the particular institutional and organizational landscape, and the ability to utilize solid performance measurements for deeper evaluation of learning outcomes (Salas and Cannon-Bowers, 2001).

While some of these capabilities can be learned and honed solely through experience, the review implies that effective training requires some degree of formal professional development-focused pedagogical foundations. In academic settings, the integration of structured team science training into existing undergraduate science curricula necessitates pedagogical changes among teaching faculty who may lack adequate training, sufficient time, or sufficient incentives (Brownell and Tanner, 2012) to recognize the value of training new scientists in effective teaming beyond basic professional behavior. Considering that the majority of academic faculty have had little or no formal pedagogical training in general, much less academic credentials in higher education or professional development, the issue of who should train may not simply be a matter of qualifications, but of supply. Similar issues arise when it comes to the identification, experience, and training of mentors. Mentorship forms a crucial relationship for the successful team scientist as well as contributes to the collaborative culture which facilitates organizational readiness. Table 1 provides a Macro-Exo-Meso-Team (MEST) summary with recommendation for implementation of developmental training norms necessary for organizational readiness.

**Table 1:** Summary and recommendations for developmental training.

**MEST level**	**Training focus**	**Key challenges identified in text**	**Developmental training strategies**	**Illustrative examples/ mechanisms**
Macro (education systems, policy, workforce norms)	Field-wide expectations for scientific preparation and careers	•Science socialized as primarily individual achievement •Lack of team science in K−12 and national standards •Absence of validated competency standards •Rapid technological and epistemic change	•Normalize team science as a core scientific competency across career stages •Develop field-level competency frameworks and assessment standards •Promote “T-shaped” scientist models balancing depth and breadth	•NASEM team science recommendations •Translational science competency frameworks •Workforce-aligned training expectations
Exo (institutional/ organizational)	Curricula, incentives, infrastructure for training	•Rigid curricula and grading structures dissuading science careers •Misalignment between collaboration demands and educational incentives •Limited pedagogical training for faculty •Resource and cost-effectiveness constraints	•Integrate structured team science training into STEM curricula •Incentivize faculty participation in team science pedagogy •Institutionalize collaboration-supportive learning ecosystems	•Curriculum redesign •Faculty development programs •Institutionally supported experiential learning
Meso (programs, learning environments, mentorship systems)	Where and how team science learning occurs	•Overreliance on “learning by immersion” combined with “sink or swim” expectations •Inconsistent experiential quality across teams •Lack of intentional reflection and feedback •Fragmented mentorship practices	•Combine experiential learning with structured reflection •Embed team science training within research programs •Leverage mentorship, peer learning, and simulation-based training	•Collaborative undergraduate research programs •Mentored team placements •Workshops, simulations, short courses
Team (individual learners, trainers, mentors)	Skill acquisition, identity formation, and practice	•Novice confusion from mixed messaging about individual vs. team science •Variable trainer experience and bias •Limited opportunities for deliberate practice and feedback	•Emphasize core, transferable team science competencies •Use evidence-based training design (practice, feedback, reflection) •Balance trainer experience with pedagogical grounding	•Skill demonstrations and practice cycles •Reflective feedback loops •Trainer development informed by learning science

## 4 Mentoring future team members

In this section, we explore the significance of mentoring in the overall process. It is essential to understand that individuals within the institution contribute toward organizational readiness. Mentoring serves a crucial function in training the next generation of scientists. For mentoring to be truly effective, there must be a clear understanding of the mentoring relationship dynamics, the pathways to be pursued, and the competencies to be developed.

### 4.1 Individuals as team drivers

Teams are inherently driven by individual aspirations and the potential for noteworthy outcomes. However, as we have seen, organizations will often prioritize individual accomplishments over collective success. This misalignment stifles the progress of team science, which is essential for achieving meaningful and real results. For organization readiness to be in place and truly flourish, it is essential that every team member understands and embraces their role in the group. The authors recognize that this speaks to institutional reward systems and the tug of war that occurs between acknowledging an individual vs. a team. This is an issue that is not easily reconciled.

Mentoring plays a transformative role in these interconnected fields by fostering collaboration and supporting individual growth. We are encouraged to prioritize a culture that values teamwork and the synergies that arise from diverse perspectives so we can tackle the complexities of various grand challenges that exist. By embracing a holistic perspective, teams can propel toward greater achievements in shared endeavors. This would be an example of the sum of the parts being greater than the whole.

### 4.2 Understanding the theoretical frameworks

Zachary looks at ways of creating a mentoring culture within an organization. The writers provide an analysis of what it requires to build a rapport for practitioners who are genuinely interested in creating a positive workplace. This rapport considers relationships and the investments made by institutions. Investments include understanding the needs of individuals to the organization or team and actively providing opportunities for training to take place (Zachary, 2005). This can include peer-to-peer mentoring, as well as having a senior person mentoring a more junior individual. Understanding the needs allows for better matching expectations and could lead to long-term relationships and collaborative opportunities. Research shows and Chan's work (2006) notes that in workplaces common complaints include poor or limited career development, a severe misalignment of expectations, and few training opportunities (Chan, 2006). These complaints are often from fresh graduates or new organization hires who are disappointed and often leave the organization feeling disenfranchised and disappointed. When turnover takes place, this leads to organizational loss and no return on the investment made. The perpetuation of this within the system is a flaw—a systemic flaw that can be reduced and even eliminated with a different level of commitment.

Organizational readiness can be linked to organizational learning and adaptability. Organizations must be ready and willing to do the work that is necessary to promote success for the individual, which would impact the team. The work of (Caci et al. 2025) builds on the work of (Zachary 2005). If the goal is to promote a mentoring culture of success then there must be deliberate actions or inputs to this end. “Creating a mentoring culture begins by engaging in a discovery process to solidly ground the work of mentoring. The discovery process focuses on understanding the dynamics of an organization's culture from multiple perspectives and determining its cultural readiness for mentoring.” (Zachary, 2005, p. 15) Developing a culture for mentoring must be deliberate if an organization is to actively demonstrate its readiness. (Zachary 2005) continues to explain: “[The] discovery [process] includes raising cultural consciousness, mapping the culture, understanding cultural ecology, identifying cultural anchors, establishing the learning anchor, testing for cultural congruence, and deciding to move forward” (p. 15).

Mapping an organization's culture looks at what the institution “pays attention to” (Zachary, p. 17). Mapping the culture paints a picture of the organization as a whole. Mapping an organization's culture is complex and nuanced. (Schein 1999) notes that this challenge is enormous, yet something to be embraced because it influences every level of the organization. Yet another aspect to be consider centers on understanding cultural ecology. This component looks at what is happening in the background of the culture. These clues tend to provide other hints as to what is happening in or impacting the organization, both internally and externally.

When considering identifying cultural anchors, it is important to understand their roles.

According to (Zachary 2005, p. 22), “cultural anchors are the moorings of an organization's culture to which a program, process, or initiative is tethered. Without strong cultural anchors, an organization may embark on or engage in organizational mentoring but drift away from its intended purpose. Mentoring, when unconnected to something larger than itself, gets buffeted by changing tides and sometimes even cast out to sea.” These anchors must engage in the process of learning which grounds the work they are doing within the organization. For mentoring to be impactful, it must be rooted on the learning of the individual, the organization and we propose the team. Learning must consider systems thinking, process improvement, and strategy. Each of these items lends itself to a stronger organization.

The next consideration entails testing for cultural congruence. Once the anchors have been identified and the learning is taking place, it becomes necessary to test for learning outcomes. As the organization grows, so does its consciousness and testing for congruence examines the fit of the learning that is taking place. This stage helps to identify if changes need to be made. The final stage outlines making decisions to move forward. Mentoring and its corresponding infrastructure must be in sync with the culture. Organizational leaders must know the direction they are heading in and develop a plan and strategy to get there. This helps guide the decision-making process and aids in determining if and how to move forward.

(Behar-Horenstein and Prikhidko 2017) examine the various context in which team science can be expanded and remain successful. They investigate how the team science framework promotes collaborations and the development of independent researchers. Here, they look at the perspectives of both mentors and mentees in a postdoctoral training program such as the T32 (Behar-Horenstein and Prikhidko, 2017). The T32 funding mechanism is an institutional National Research Service Award (NRSA) grant from the National Institutes of Health (NIH). The program is designed to prepare pre- and post- doctoral trainees for careers in health fields by providing various types of support. The goal is to train the next generation of researchers for careers that will significantly impact the nation's health needs. Institutions and their systems serve as the backdrop for how individuals are trained.

If specific skills (outcomes) are desired in the next generation of researchers, clinician, educators, or employees, we must closely look at what the inputs (skills taught and learned) are as mentioned earlier. Mentors and mentees can directly impact how the outcomes are derived. (Behar-Horenstein and Prikhidko 2017) found that mentees valued the opportunity to work with others. Having the opportunity to engage with others, in different departments, was viewed as positive. These opportunities were seen to broaden the mentees' perspectives and improve their research capabilities. Additionally, being able to collaborate was critical to the mentee's growth and development (pp. 9–11).

As noted in Section 1.1, the guiding theoretical framework rests in organizational readiness. Organizational readiness is closely related to learning and adaptability, which requires commitment to actions that foster individual and team success. To build a mentoring culture, organizations must engage in deliberate discovery process that examines cultural dynamics and readiness. Creating a mentoring culture demands intentional efforts to align practices with mentoring and team success goals. Having a mentoring infrastructure that rewards both the individual and corresponding teams will advance the capabilities of the organization.

### 4.3 Team science as a mechanism for instruction and change

One of the outcomes of good mentoring is seen in the description of how multidisciplinary interactions helped to foster new ideas and approaches. Some mentors were deliberate in establishing and promoting a team culture. For these mentors, communication, teamwork, and shared responsibility were emphasized. These mentors demonstrated the skills that were necessary to be a successful independent researcher. However, these mentors also spent time emphasizing what it meant to be a member of a successful team. Successful mentors actively demonstrated the skills that were necessary to promote an inviting and belonging environment where individuals could grow, and where teams could succeed. Spaces included classrooms, faculty lounges or other spaces, and laboratories where different types of interactions could take place. This afforded the mentors the opportunity to see and experience different types and levels of interactions (Behar-Horenstein and Prikhidko, 2017). The bottom line—working in teams to foster new and different ideas and approaches is a necessary skill for today and tomorrow. This research shows that the team science framework can be beneficial to mentees by allowing them to engage with different colleagues which broadens their perspectives and enhances their research quality. Higher quality researchers impact the standing of institutions.

### 4.4 Elements of effective mentoring relationships

(Banack 2020) explains how mentoring serves as a catalyst for early to mid-career researchers. The author describes how being mentored in multidisciplinary environments is beneficial to personal and team-based long-term goals and objectives. Mentoring systems within an institution that promote both individual and team-based projects and research can serve as a career development tool. Individually, researchers seeking to develop productive and well-funded research programs tend to benefit from mentoring. Having the opportunity to work in multidisciplinary teams can also lead to productive and well-funded research programs which impacts the institution's bottom line.

An important aspect of organizational readiness should consider the role of mentoring and the systems that are in place to promote effective mentoring. (Banack 2020) identifies five essential dimensions that make mentorship effective. These dimensions are (1) advice, (2) interpersonal connections, (3) opportunities, (4) protection, and (5) resources. In this framework, mentors provide mentees with both professional and personal advice. This includes career advice (i.e., job search process), research-related advice (e.g., proposal development and submission) and personal advice (e.g., work-life balance). (p. 4) The advice provided should be aligned with the mentee's goals and aspirations.

Another aspect of a good mentoring relationship is linked to interpersonal connections, which serves as the basis for the relationship. “Connection” is seen in the acceptance, empathy, respect, and trust that is displayed overall in the relationship (Banack, 2020, p. 4–5). Effective mentoring leads to a strong relationship where values are central. The third dimension mentioned by (Banack 2020) centers on mentors providing mentees with opportunities. These opportunities include working on different types of projects, developing questions and protocols and doing data analysis. Additional opportunities include working on peer-reviewed manuscripts and having professional development opportunities to attend conferences and meetings and giving talks. Protection within this sphere includes mentors advocating for mentees in professional disputes to help find resolutions. Through this lens, mentors serve as guides through various experiences (p. 4). Access to resources is the fourth dimension that mentors can help to facilitate. When the mentor is unable to provide access to resources, they should be able to advocate for resource access when possible.

The components listed collectively can support a strong mentoring relationship. At the foundation level is the emphasis on interpersonal connections. (Banack 2020) highlights acceptance, empathy, respect, and trust as core characteristics needed for mentoring relationships to succeed. These items can help to create psychological safety which allows mentees to ask questions and acknowledge gaps in their knowledge without fear of judgment. This core emotional underpinning makes the relationship resilient and sustainable over time.

Second to the resilience of any mentoring relationship is values. When mentors and mentees take the time to discuss and revisit values explicitly, it helps to shape and expand identities. Additionally, this helps to promote long-term goals that are grounded in ethical practices and organizational processes. This moves the mentor-mentee to one of a formative partnership vs. one that is merely transactional.

The modeling of behavior is salient to the mentoring relationship. The third aspect discussed by Barnack focuses on the mentor actively supporting the mentee's growth. A professional development or growth plan can include having the mentee work on projects, presentations, and various types of publications. By affording the mentee these opportunities, the mentor demonstrates trust which leads to good will.

With the mentor showing trust and visibly guiding the mentee, this helps the mentee interpret challenges, make informed decisions, and learn from setbacks. This active behavior acknowledges competence building through active learning, contextual understanding, and reflection. Within this relationship, the mentor serves as a resource, helping to facilitate new opportunities and bridge gaps. The elements outlined show an interconnectedness that builds a holistic framework that can blossom in an organization that is ready for change and growth.

### 4.5 Organizational readiness and competencies

Organizational readiness depends on the capabilities of the individual—the mentor and mentee to be engaged in a mutually beneficial relationship. For the ecosystem (institution) to thrive, the system must be constructed in such a way that there is a continuous loop of inputs based out outcomes. The inputs are the competencies and skill set that appear in the mentoring relationship. The work of (Shah and Fiore 2022) look at what is required to build effective mentoring teams using team science competencies. These competencies apply to science and non-science workers alike as they help to make a more effective and well-rounded individuals, researchers, or team members—ultimately, a more prepared institution.

Competencies that are linked to mentoring teams include active listening, assertive and non-verbal communication, coordination, collaborative orientation, and cultivating appreciation for varied perspectives. These competencies are not new- they serve as the basis for good relationships. We support listing of competencies and contend that instead of focusing on the novelty of the skill, we should focus on the mastery of the skill (Shah and Fiore, 2022).

If individuals are failing at the foundational (most-basic) level to demonstrate various skills or competencies, the organizations that they are a part of should re-evaluate their readiness plan(s). Organizational readiness is systemic—there is a constant cycle of inputs and outputs which determines how success looks. Poor, flawed, or low-quality inputs at the foundational level should be addressed as early as possible as they greatly impact growth, innovation, and sustainability issues (desired high-quality outputs) for the individual and organization. Table 2 provides a Macro-Exo-Meso-Team (MEST) summary with recommendation for implementation of mentoring norms necessary for organizational readiness.

**Table 2:** Summary and recommendations for mentoring.

**MEST level**	**Mentoring focus**	**Key challenges identified in text**	**Mentoring & readiness strategies**	**Illustrative examples/mechanisms**
Macro (disciplinary norms, workforce expectations, funding models)	Mentoring as a workforce-development and scientific norm	•Persistent privileging of individual achievement •Inconsistent expectations for mentoring quality •Limited recognition of mentoring as scientific labor	•Elevate mentoring as a core component of scientific productivity •Align mentoring expectations with team science outcomes •Encourage funding mechanisms that support structured mentoring	•NIH T32 and NRSA training grants •National calls for mentoring accountability •Field-wide mentoring competency frameworks
Exo (institutional/ organizational ecosystem)	Organizational readiness and mentoring culture	•Misalignment between institutional priorities and team-based mentoring •High turnover from unmet developmental expectations •Lack of deliberate mentoring infrastructure	•Conduct organizational “mentoring readiness” discovery processes •Institutionalize mentoring cultures through policy and investment •Align mentoring systems with learning and adaptability goals	•Zachary's mentoring culture discovery process •Formal mentoring programs and matching systems •Incentives including organizational wide recognition for mentoring excellence
Meso (programs, teams, mentoring networks)	Mentoring relationships within team and program contexts	•Inconsistent mentor–mentee expectations •Limited exposure to interdisciplinary collaboration •Variable modeling of team science behaviors	•Clarify mentoring pathways, roles, and competencies •Use team science as a mechanism for instruction and culture change •Embed interdisciplinary engagement within mentoring structures	•Team-based postdoctoral programs (e.g., T32) •Peer and near-peer mentoring models •Multidisciplinary project teams
Team (mentor–mentee dyads and individuals)	Skill mastery, identity formation, and relational competence	•Foundational competencies unevenly developed •Mentoring relationships lacking trust or clarity •Overemphasis on novelty over mastery	•Develop and assess core mentoring and team science competencies •Emphasize mastery through repetition and feedback •Model effective team behaviors through mentoring	•Banack's five mentoring dimensions (advice, connection, opportunity, protection, resources) •Competencies such as active listening, coordination, and collaborative orientation

## 5 The professionalization of science teams

As collaboration science has intensified—organizationally, internationally, and interdisciplinarily—scholars have begun examining not only how teams function, but how team-based science itself becomes professionalized. Professionalization refers to the development of codified norms, recognized expertise, formal roles, and institutional support that collectively define a field as an occupation with standards and legitimacy (Mieg and Evetts, 2018). Applied to science teams, professionalization involves building consistent skill sets, delineating responsibilities, creating new career pathways, and embedding team-based work into organizational structures and cultures. Across varied contexts—from university research administration to STEM peer leadership, science communication, international collaboration, and citizen science—team science is becoming a site of professional identity formation and occupational differentiation.

A further complication on the matter where team science is learned relates to the “professionalizing” of the field. As it stands, Team Science as a subject is largely considered an interdisciplinary endeavor (LeDee et al., 2011; NASEM, 2025)—though it has yet to be determined whether it has become pedagogically flexible enough to achieve true interdisciplinary integration (O'Rourke et al., 2019). And can it evolve to transdisciplinarity? That is, can team science develop an identity that has synergized its component disciplinary epistemologies into something new and with a truly unique professional vocabulary? It may be that Team Science as a field is on a trajectory for such an achievement, but the systemic complexity of an interdisciplinary learning environment is further compounded and complicated by the presence of team science within the often-competing ecosystems of industry and academia. Scientific research has historically navigated its progress often through mutually beneficial collaborative agreements with industry. The production of research output, however, is not comparable to the production of team capability. Partnerships with industry and academia can be created to monetize or patent research output; but such partnerships are based on the mutual benefits of commercial success—not on the shared development of what in industry would be considered human resources. Successful teams are an essential engine of business success—in some sense, the “secret sauce” of industryas in Google's “Project Aristotle,” undertaken, between 2012 and 2014, was a large, multidisciplinary study of 180 of their own teams using hundreds of team attributes (Duhligg, 2016). They began with some strong presuppositions about what made successful teams (e.g., having the smartest or highest performing individuals; matching or complementing temperaments; experienced managers, etc.) but were surprised to see that what really was common among the most successful teams were things like psychological safety, dependability, role clarity, meaningful work, and belief that team members' efforts were making an impact. If one of the largest and most ubiquitous businesses in the world was only recently able to gain an understanding of team efficacy within their own organization, it may be that professionalization of team science in the industrial space has a bit further to go than academia, where research scientists from all disciplines are trained prior to roles in industry. A singular recognized set of standards, competencies, and approaches for team success shared by industrial research teams and academic research teams may be a long way from being reconciled. However, the common denominator of most research teams—industrial or academic—is formative training in a higher education setting. In a deeper sense, the common denominator of scientific teams may truly be the formative experiences constituting their earliest engagement in science education and how it prepared or hindered that next step on their scientific career trajectory. But it is in higher educational environments where the pursuit of scientific careers begins their recognizable patterns. To centralize formal team science training in higher education would provide developing scientists critical exposure to team science training opportunities that are crucial as their professional career progresses.

### 5.1 From the individual investigator to the professional team scientist

The historical image of the “sole investigator” persists culturally, yet the realities of scientific work increasingly require coordinated expertise across multiple disciplines, roles, and institutions. Leahey's analysis traces how scientific practice has shifted structurally and symbolically toward teamwork, documenting the rise of large-scale collaborations, co-authorship networks, and multi-institutional research groups. As science becomes more collaborative, professional roles must evolve science workers to require specialized interpersonal, organizational, and communicative competencies that differ from those traditionally associated with disciplinary scholarship (Leahey, 2016).

This shift necessitates the institutionalization of new expectations about how scientific labor is organized. Yet, professionalization emerges as science workers acquire consistent training in team-based competencies, organizations formalize collaborative procedures, and interdisciplinary work becomes normatively valued. Yet this transition also introduces challenges: scientists must navigate evolving norms around authorship, leadership, collective credit, and cross-disciplinary translation of knowledge.

Professionalization, then, is not merely an outcome—it is an ongoing process through which norms, roles, and legitimacy structures coalesce around new forms of scientific practice (Mieg and Evetts, 2018) emphasizing life course competency building (Lotrecchiano et al., 2020). The movement toward team-based science is inseparable from the professional systems designed to support it.

### 5.2 Professional roles in organizational contexts

Because team science involves actors embedded in institutions, many of its professionalization dynamics unfold in organizational settings. Schäfer and Fähnrich argue for an “organizational turn” in science communication research, noting that communicating science—from within research teams to external stakeholders—is shaped by organizational mandates, cultures, and expectations (Schäfer and Fähnrich, 2020). In team science, communication is not merely a skill; it becomes a formalized responsibility and source of expertise. Researchers must navigate institutional policies, external engagement demands, and organizational communication infrastructures—competencies characteristic of an emerging profession within scientific organizations.

Similarly, (Windsor 2021) demonstrates how interdisciplinary computational communication science requires professional bridging roles that can translate across disciplinary languages, methodologies, and epistemologies. Such work depends heavily on professional identities that are hybrid in nature: neither strictly disciplinary nor purely administrative but integrative and boundary-spanning (Boardman, 2011; Golden and Veiga, 2005). Professionalization thus involves developing and legitimizing these hybrid identities so they can effectively support interdisciplinary teamwork (Windsor, 2021).

These organizationally anchored roles illustrate that team science evolves through processes that codify responsibilities, build standard practices, and elevate forms of expertise. As organizations develop clearer expectations for how collaboration should function, they contribute to the professional infrastructure of team-based research.

### 5.3 Research management and administrative professionalism

Team science relies not only on researchers but on a growing cadre of research management professionals. As (Acker et al. 2019) note, university research administrators—once viewed as purely bureaucratic—are increasingly recognized as practitioners with specialized knowledge, strategic expertise, and a critical role in enabling complex research collaborations. Their professionalization occurs through formal training, occupational identity development, and integration into the strategic core of research institutions (Acker et al., 2019).

Globally, similar professionalization trajectories appear. Research management thus represents a profession emerging in parallel with team science, ensuring that teams receive the administrative, logistical, and regulatory support necessary to function effectively. Without such professional roles, large-scale collaborations would struggle to meet compliance requirements, manage international partnerships, or navigate complex funding landscapes.

### 5.4 Expertise, skill development, and identity formation

Professionalization also requires that individuals develop and internalize the specialized expertise needed to function effectively within science teams. Several bodies of work illustrate how identities and competencies are shaped through practice and formal institutional efforts.

Bowling examines peer leaders in STEM education, showing how professionalizing these roles—through training, evaluation mechanisms, and formal recognition—enhances collaborative learning environments and supports more effective team-based instruction. In science teams, similar professional development processes help build competencies in communication, conflict resolution, distributed leadership, and collaborative problem-solving (Bowling, 2015).

In a study of medical residents, Phillips and Dalgarno highlight a related dynamic: professional expertise is not limited to technical skills but includes compassion, relational abilities, and the capacity to work effectively within collaborative clinical environments. Translating this insight to research teams underscores that scientific professionalism increasingly hinges on interpersonal and social expertise as much as disciplinary knowledge (Phillips and Dalgarno, 2017).

McLain also shows how identity construction among informal science educators depends on institutionalized pathways, community norms, and self-perception as professionals (McLain, 2017). This mirrors processes occurring in team science, where researchers increasingly see themselves not only as disciplinary experts but also as collaborators, communicators, and co-creators of knowledge.

Together, these studies illuminate how professional identities are socially constructed through training, practice, and organizational recognition—and how team science relies on cultivating these identities to sustain collaborative engagement.

### 5.5 Internationalization, collaboration, and professional norms

The globalization of science has intensified team-based work across national boundaries, creating new layers of professionalization. Dusdal and Powell document the motivations, benefits, and challenges of international collaborative research, emphasizing that successful cross-border teams depend on shared norms, trust-building practices, and professional competencies that facilitate collective work despite differing institutional cultures (Dusdal and Powell, 2021).

International collaborations require scientists to develop expertise in coordination, intercultural communication, and the management of diverse regulatory and organizational environments. These competencies become formalized through professional standards, training programs, and institutional guidelines, contributing to a global professional culture of team-based science.

Moreover, as Mohajerzad et al. show, collaboration between scientists and practitioners influences not only research processes but also how findings are perceived, utilized, and legitimized (Mohajerzad et al., 2021). This underscores that professionalization in team science extends to building shared expectations about evidence standards, relevance, and applicability—especially when teams include stakeholders outside academia.

### 5.6 Professionalization through innovation and new knowledge ecosystems

The professionalization of science teams is intertwined with broader shifts in innovation and knowledge production. Robbins and O'Connor identify similar dynamics in the evolution of innovation management as a profession: roles become more formalized as organizations recognize the need for specialized expertise to coordinate cross-functional teams, manage uncertainty, and foster collaboration. Science teams operate under analogous conditions, where interdisciplinary innovation requires professionals who can integrate diverse inputs and strategically steer collaboration (Robbins and O'Connor, 2023).

Similarly, (Strasser and Haklay, 2018) analyze the role of citizen science, showing how expertise in participatory methods, data quality management, and collaborative knowledge production is becoming professionalized. Citizen science teams challenge traditional hierarchies of expertise, yet still require consistent standards, training, and organizational frameworks to function effectively. As citizen participation becomes woven into scientific research, new professional roles emerge to bridge experts and publics.

Friederich and Schelle offer a broader theoretical perspective: professionalization models based on evidence and efficiency can improve practice by codifying standards and optimizing processes. Applied to science teams, such models can enhance collaborative productivity, research quality, and organizational learning (Friederich and Schelle, 2021).

### 5.7 Synthesizing professionalization across contexts

Across these varied literatures, several themes consistently emerge that define what it means for science teams to become professionalized:

- **Training and Competency-Building**: Researchers benefit from structured development of “team science skills,” including effective communication, coordination, conflict resolution, leadership, data sharing, and collaborative decision-making. Such training has been shown to improve team performance and research productivity across disciplines (Lotrecchiano et al., 2023; Stokols et al., 2008b).
- **Mentorship and Role Development**: Mentoring, particularly for early-career scientists, fosters the adoption of teamwork as an integral component of professional identity rather than an occasional task. Effective mentorship can help emerging investigators navigate collaborative dynamics, assume leadership roles, and internalize team-based norms (Lotrecchiano et al., 2021; Pfund et al., 2016).
- **Recognition of Team-Specific Contributions**: Traditional metrics often emphasize solo-authored publications, overlooking crucial collaborative contributions such as data curation, coordination, facilitation, and shared authorship. Explicit recognition of these roles encourages meaningful engagement in team science and supports career advancement within collaborative contexts (Börner et al., 2010; Wuchty et al., 2007).
- **Cultural Shift in Research Careers**: Advancing team science requires reframing the career narrative to value interdisciplinary collaboration alongside independent investigation. Recognizing collaborative work as a legitimate, prestigious pathway can help shift institutional reward systems and support sustainable engagement in large-scale, integrative research (Hall et al., 2012).

Professionalization, therefore, is not a singular process but a multilayered transformation occurring across individual, organizational, and systemic levels. Team science becomes sustainable when the roles, responsibilities, competencies, and identities that support collaboration are recognized, codified, and institutionally rewarded.

As scientific challenges become more complex, the professionalization of team science becomes essential for producing robust, socially relevant, and innovative knowledge. The literature illustrates that professionalization is multifaceted: it involves cultivating specialized expertise, formalizing roles within organizations, developing shared norms across global networks, and legitimizing hybrid identities that bridge disciplinary and sectoral divides. Without these professional structures, science teams would lack the stability, clarity, and institutional support necessary to function effectively in contemporary research ecosystems.

Team science is not merely a method of organizing research—it is a profession in the making. Its professionalization reflects broader transformations in how expertise, collaboration, and innovation are conceptualized in the 21st century. Therefore, as career identities change so must institutions. Professionalzing team workers and institutionalizing team science go hand in hand but different dimension that need to be considered. Table 1 outlines these differences. Table 3 provides a Macro-Exo-Meso-Team (MEST) summary with recommendation for implementation of professionaling norms necessary for organizational readiness.

**Table 3:** Summary and recommendations for professionaling team science.

**MEST level**	**Professionalization focus**	**Key dynamics & challenges identified**	**Professionalization strategies**	**Illustrative roles, mechanisms, or examples**
Macro (field, global, policy, and workforce systems)	Legitimizing team science as a profession	•Persistence of the “sole investigator” norm •Fragmentation across disciplines, sectors, and nations •Lack of shared global standards for team competencies •Tension between academic and industrial ecosystems	•Codify team science norms, competencies, and standards •Promote team science as a legitimate career pathway •Align academic, industrial, and international expectations	•National and international competency frameworks •Global collaboration norms •Recognition of team science as an occupational field
Exo (Institutional/ Organizational)	Embedding professional team roles in organizations	•Misalignment between collaborative labor and reward systems •Undervalued hybrid and boundary-spanning roles •Limited institutional recognition of team-based expertise	•Institutionalize collaborative procedures and expectations •Formalize hybrid professional roles •Reward team-based contributions in evaluation systems	•Research administrator professionalization •Science communication roles •Interdisciplinary program structures
Meso (programs, teams, networks, and knowledge ecosystems)	Operationalizing professional roles and practices	•Ambiguity in authorship, leadership, and credit •Coordination challenges in large-scale and international teams •Inconsistent mentoring and training pathways	•Standardize team practices and role clarity •Integrate mentorship and training within team structures •Support boundary-spanning and integrative functions	•Team charters and role definitions •Mentored interdisciplinary teams •International collaboration guidelines
Team (individuals, roles, and professional identity)	Skill development and identity formation	•Transition from disciplinary to collaborative identity •Underdeveloped interpersonal and organizational skills •Limited recognition of relational and coordination labor	•Develop mastery of team science competencies •Foster professional identity as a team scientist •Emphasize mentorship, reflection, and practice	•Competencies in communication, coordination, conflict resolution •Distributed leadership roles •Identity as collaborator, integrator, or facilitator

## 6 Institutionalizing team science

Because team science introduces new dynamics (interdisciplinary communication, shared credit/data, complex coordination), sustaining it often requires more than goodwill—team science needs deliberate structures, training, and incentives. That's where institutionalization comes in. It is important to recognize that though similar to professionaling team science, there are definitive differences between professionalizing and institutionalizing team science and what change is necessary (Table 4).

**Table 4:** A comparison of professionalizing vs. institutionalizing in team science.

**Dimension**	**Professionalizing**	**Institutionalizing**
Focus	Individuals/teams (skills, training, identity)	Organizations/institutions (policies, infrastructure, culture)
Goal	Build competent, team-ready scientists and teams.	Embed and sustain team science as a structural norm
What changes	Training curricula, mentoring, team norms, leadership practices, authorship norms	Tenure & promotion criteria, administrative support, resource allocation, institutional policy, shared infrastructure
Who drives it	Scientists, team leaders, mentors, trainers	Department/institution leadership, funding agencies, research offices
Type of change	Cultural and behavioral within teams	Structural and organizational across institutions

From an industrial-organizational psychology perspective, an organization's reward system is a decisive contextual factor that shapes employee motivation (Aycan, 2000; Furtado et al., 2012; Van Eerde, 2015). Work motivation, in the classic definition by Latham and Pinder, is a set of internal and external forces that Initiate work-related behavior and determine the form, direction, intensity, and duration of those behaviors (Latham and Pinder, 2005). This motivation is observed through a dynamic, goal-directed, resource-allocation process (Kanfer et al., 2017), meaning individuals allocate their personal resources—such as attention, effort, and time—to shape the direction and persistence of their work activities allocate their personal resources—such as attention, effort, and time—to affect the direction and persistence of their activities at work. The resources an individual allocates is influenced by the environment (Latham and Pinder, 2005). Herein lies the central conflict for institutionalizing team science: the academy presents a case of systemic misalignment. The stated goal of collaboration is undermined by the very context that provides opportunities for and constraints on collaboration (Latham and Pinder, 2005). While institutions and funders increasingly advocate for large-scale, translational collaboration, their core reward and incentive structures remain overwhelmingly rooted in individualistic metrics (Klein and Falk-Krzesinski, 2017). Promotion, tenure, and prestige are still primarily and most easily calculated through publication count, but more critical to this conversation, from the implied hierarchy of value defined by being first or senior author on publications and filling the formal role of Principal Investigator (PI) on grants. This creates a psychological and motivational conflict in which what the individual is being asked to do (e.g., collaborate) is not what the environment rewards. In other words, the collective effort e effort required for team science is structurally disincentivized in favor of individualistically led activities that have a clearly defined career path.

### 6.1 Key features of institutionalizing team science

- **Incentive Structures Aligned to Team Science Contributions**: Institutions can promote effective team science by adapting promotion and tenure policies to recognize a broader range of contributions. This includes authorship in interdisciplinary or non-traditional journals, non-traditional roles such as coordination or facilitation, data sharing, and contributions to process-oriented outcomes (Klein and Falk-Krzesinski, 2017). Aligning incentives with collaborative work ensures that team-based achievements are valued alongside traditional individual metrics.
- **Administrative, Infrastructural, and Resource Support**: Successful team science often requires robust institutional infrastructure. Shared data platforms, administrative support for coordination, resources for ethics and compliance, and dedicated staffing are critical, particularly for large-scale or multidisciplinary projects. Providing these supports reduces barriers to collaboration and enhances the efficiency and impact of team-based research.
- **Embedding Team Science into Institutional Norms and Evaluation Systems**: To normalize collaborative research, institutions should treat team-based efforts not as exceptions or add-ons, but as a mainstream and expected mode of operation. Incorporating team science into standard evaluation frameworks signals its legitimacy and encourages consistent engagement by faculty and researchers.
- **Long-Term Sustainability and Structural Support**: Many team science initiatives evolve into large, persistent, multiteam systems. Sustaining these complex collaborations requires stable organizational support to manage coordination, translation of findings, and data sharing across projects. Without such structural backing, collaborative efforts risk fragmentation or failure despite high potential impact.

### 6.2 The need for appropriate reward structures

To institutionalize team science, the academy must fundamentally disrupt the entrenched reward structure that privileges the artifacts of individualism over the complex process of collaboration. This reliance on singular roles functions as a critical environmental constraint (Latham and Pinder, 2005), preserving an outdated lone genius paradigm that renders invisible the essential, specialized contributions of methodologists, data curators, and other essential team members. However, this devaluation of collective effort is compounded by a pervasive culture of internal competition for limited institutional resources. Because work motivation is fundamentally a process involving finite attention and effort (Kanfer et al., 2017) scientists are forced to rigorously assess the utility of every investment. In a zero-sum environment where colleagues compete for recognition and funding, the institutional mandate to collaborate collides with the financial pragmatics of survival. Scientists are structurally discouraged from sharing data or investing time in a colleague's project when that same colleague is their primary competitor for departmental resources or funding awards. These dynamics turns career viability into a struggle for professional survival, effectively squashing the desire to form teams because working alone becomes the most rational strategy for keeping one's job within the existing organizational context (Kanfer et al., 2017).

A practical pathway to disrupting these norms lies in creating and adopting new metrics for evaluation that promote collaboration. The question for assessing readiness for promotion is no longer how many publications might you have as first author, but rather, what specific contribution(s) did you make to catalyze the team's success? This shift requires a granular evaluation of the nature of the contribution, recognizing that scientific impact can occur at any phase of the translational research lifecycle (Hall et al., 2018; Lotrecchiano et al., 2025; Trochim et al., 2011), from the initial spark of ideation and hypothesis generation to the critical downstream phases of methodological design, data stewardship, or translational application. By valuing the specific expertise that enabled the work, rather than just the final authorship order, institutions can legitimize the diverse forms of intellectual labor required to solve complex problems. This may involve the adoption of frameworks like the Contributor Roles Taxonomy (CRediT), which moves beyond authorship order to specify 14 distinct roles (Allen et al., 2019; Brand et al., 2015). By requiring such taxonomies, institutions provide Promotion and Tenure committees with the concrete data needed to evaluate a faculty member's specific role, replacing ambiguous, subjective judgments with transparent evidence of contribution. This enables the construction of a career narrative defined by holistic scientific impact, rather than one constrained by the rigid hierarchy of authorship order.

Ultimately, the institutionalization of team science requires a holistic cultural shift driven by leadership across organizational levels. While aligning financial incentives is critical, institutions must also actively cultivate a climate of collaboration through intentional hiring and supporting team science related professional development. This means expanding recruitment criteria beyond the traditional scientific expertise signified by the PhD credential to prioritize candidates who have demonstrated the specific acumen required to lead, co-lead, and mentor within complex research teams. Once hired, faculty members must be supported by an infrastructure that rewards the process of team formation and provides training in the essential skills of collaboration, not just rewarding the final products that result from teamwork like grants and publications. As funding agencies increasingly mandate and reward these behaviors (e.g., by recognizing data stewardship or community partnerships as translational milestones), universities must respond by ensuring their faculty possess not only the intellectual capability to solve problems but the relational capability to function as effective team members. By synchronizing hiring practices, skill-building investments, and formal reward structures, the academy can finally move team science from an ideal to a normative, pragmatic, and valued pathway for a successful scientific career. Table 5 provides a Macro-Exo-Meso-Team (MEST) summary with recommendation for implementation of institutionaling norms necessary for organizational readiness.

**Table 5:** Summary and recommendations for institutionalizing team science.

**MEST level**	**Institutional strategies**	**Key dynamics and challenges**	**Institutionalization strategies**	**Illustrative roles, mechanisms, or examples**
Macro (field/policy/ funding ecosystem)	Align funding priorities, research evaluation norms, and national science policy with collaborative research models	Misalignment between policy-level encouragement of collaboration and academic reward systems rooted in individual achievement; persistence of the “lone-genius” paradigm	Develop contribution-based impact metrics across the translational research lifecycle; require transparency in reporting collaborative contributions	Translational science evaluation frameworks; funding agency collaboration requirements; contributor-based reporting standards
Exo (inter-organizational/ multiteam systems)	Provide sustained coordination structures and shared infrastructure for interdisciplinary and multi-institutional collaboration	Coordination complexity, data-sharing barriers, compliance burdens, and sustainability challenges in large-scale collaborations	Establish shared administrative systems, data platforms, and coordination support for long-term multiteam research systems	Shared data repositories; cross-institutional research centers; project coordination staff; multiteam translational initiatives
Meso (institutional/ university level)	Reform promotion and tenure criteria; embed team science into evaluation, hiring, and training systems; provide administrative and infrastructural support	Institutional reward systems prioritize PI status, first authorship, and publication counts; internal competition for scarce resources discourages collaboration	Align incentives with collaborative contributions; normalize team science within evaluation frameworks; invest in collaboration training and support structures	Promotion and tenure policy reform; collaborative hiring criteria; research development offices; compliance and coordination infrastructure; team-science professional development
Micro (individual scientist level)	Recognize diverse scholarly contributions and support collaborative skill development	Motivational conflict between collaborative expectations and individual career advancement; resource-allocation decisions shaped by institutional incentives	Evaluate faculty based on contribution-specific impact rather than authorship hierarchy; legitimize diverse forms of intellectual labor	Contributor Roles Taxonomy (CRediT); recognition of roles such as data curator, methodologist, facilitator, coordinator, and community partner

## 7 Sustaining team science

Organizational design provides the foundational architecture that enables team science to function and endure. Institutions that effectively support interdisciplinary teams typically build coordinated infrastructures—policies, shared platforms, resourcing strategies, and governance models—that align with the demands of collaborative inquiry. These infrastructures stabilize collaborative work by clarifying expectations, reducing ambiguity, and facilitating cross-boundary coordination. A key structural factor is the implementation of shared or distributed leadership models. Traditional principal investigator–centered leadership may limit creativity and reduce the agency of team members, especially in large, cross-disciplinary collaborations where expertise is distributed unevenly across fields (Aarons et al., 2020). Shared leadership fosters adaptability and empowers individuals to take the lead in areas where their expertise is most salient (Falk-Krzesinski et al., 2011). Such models have been shown to enhance innovation, communication quality, and overall team climate.

Institutional rewards and evaluation systems also shape the viability of long-term team science. Standard academic metrics often undervalue collaborative work, reinforcing the dominance of individual authorship and PI-centric grants (Hackett, 2005; Holbrook, 2010). Interdisciplinary teams require recognition systems that value coordination, integration, facilitation, data stewardship, and other forms of “invisible work” that are essential to collaborative knowledge production (Cummings and Kiesler, 2005; Garg et al., 2022). When reward systems are misaligned with collaborative practices, teams often experience diminished morale, inequity, and turnover—outcomes that undermine long-term sustainability.

Funding structures represent another critical element. Interdisciplinary and systems-oriented research often evolves iteratively, incorporating emergent questions and approaches as new knowledge is uncovered (Fernández-Giménez et al., 2019). Yet federal and institutional grant mechanisms tend to prioritize predictable timelines, discrete deliverables, and linear research pathways. Sustaining team science therefore requires flexible funding mechanisms—such as multi-stage grants, center-based support, and institutionally supported bridging funds—that can adapt to the non-linear nature of collaborative inquiry. Programs such as the NIH Clinical and Translational Science Awards (CTSA) demonstrate the power of long-term, infrastructure-level funding to stabilize collaborative networks and cultivate interdisciplinary training pathways.

Physical and virtual infrastructures also influence sustainability. Purpose-built collaboration spaces, digital communication tools, team dashboards, and interoperable data systems facilitate coordination across disciplines, institutions, and geographic boundaries (Aldrich and Al-Turk, 2017; Olson and Olson, 2014). Teams require platforms that support both synchronous and asynchronous collaboration, enabling distributed work while maintaining cohesion and transparency.

Collectively, these organizational structures establish the conditions necessary for team science to take root. Yet structural supports alone are insufficient. The persistence and success of interdisciplinary teams depend equally—if not more—on the relational and socio-cultural dimensions of collaborative work.

### 7.1 Social, relational, and cultural dynamics of sustained collaboration

Relational dynamics form the social fabric through which team science is enacted. Trust, psychological safety, interpersonal respect, and shared norms are essential to sustaining collaborative engagement (Edmonston, 1999; Salas et al., 2018). Without these elements, structural supports lose their potency, and teams are more likely to experience conflict, disengagement, and fragmentation.

Interdisciplinary collaboration introduces unique relational challenges. Team members often come from disciplinary cultures with differing values, methods, epistemologies, and communication styles (Becher and Trowler, 2001). These differences can generate misunderstandings or conflict when not explicitly acknowledged and negotiated. Maintaining long-term collaboration therefore requires intentional developmental processes such as conflict-resolution training, team charters, communication protocols, and reflective dialogue practices (Bennett and Gadlin, 2012; Bennett et al., 2014; Eigenbrode et al., 2007; Lotrecchiano et al., 2023)

Psychological safety plays a particularly powerful role. When team members feel safe to express uncertainty, challenge assumptions, and propose unconventional ideas, teams exhibit higher levels of creativity, integration, and learning (Edmonston, 1999). Psychological safety fosters the open communication necessary for interdisciplinary synthesis and reduces the interpersonal risks associated with boundary-spanning work.

Mentorship is another key relational mechanism in sustaining team science. In collaborative environments, mentorship extends beyond technical skill development to include relational competencies, disciplinary translation, negotiation of roles, and identity development as an interdisciplinary scholar (Friedman et al., 2024; Pfund et al., 2016). Effective mentorship nurtures early-career researchers' capacity to collaborate, thereby contributing to the long-term viability of the team and the sustainability of the broader team-science ecosystem.

Additionally, diversity, equity, and inclusion (DEI) are essential catalysts for sustainable collaboration. Interdisciplinary teams are most effective when they reflect cognitive diversity—distinct ways of thinking, problem-framing, and interpreting knowledge (Page, 2007). Yet diversity alone is not enough: inclusive climates that guarantee equitable participation and influence are critical for sustaining engagement and reducing attrition, especially among marginalized scholars (Nápoles et al., 2017). Teams that prioritize relational equity and inclusive communication practices tend to produce more innovative and integrative outcomes (Singh et al., 2018). In short, relational dynamics are at the heart of sustainable team science. They determine whether teams can bridge disciplinary boundaries, navigate conflict, and remain cohesive across time.

### 7.2 Knowledge integration, management, and innovation sustainability

Knowledge management is one of the most challenging—and most consequential—elements of sustaining team science. Interdisciplinary teams must integrate disparate knowledge systems, methods, conceptual frameworks, and data forms. This integrative work is cognitively demanding and logistically complex (Bromham et al., 2016; Fiore, 2008). One persistent threat to collaborative sustainability is knowledge hiding or unintentional knowledge loss. Knowledge hiding undermines trust, creates information asymmetries, and obstructs coordinated action (Liu et al., 2020). Even when not intentional, the absence of structured knowledge-sharing systems can lead to siloing, duplication of effort, and breakdowns in collective sensemaking.

Teams can mitigate these challenges through deliberate knowledge-management strategies, including:

- **Shared digital repositories** to document decisions, data, and methods (Potterbusch and Lotrecchiano, 2018).
- **Cross-training** to build mutual understanding (Blackman and Davison, 2004).
- **Boundary objects** such as models, diagrams, and shared vocabularies to support integration (Star and Griesemer, 1989).
- **Regular interdisciplinary meetings and reflection sessions**.
- **Knowledge brokers or integrators** who help translate concepts across fields (Meyer, 2010).

Innovation sustainability requires balancing stability with adaptability. Teams must establish processes that are robust enough to maintain continuity but flexible enough to incorporate new data, shifting goals, or emerging opportunities (Mintchev et al., 2022). This is the core of a complex adaptive systems approach, which positions teams as dynamic networks capable of learning and evolution (Gerrits, 2021). Leadership again plays a pivotal role in knowledge management. Leaders who promote transparency, model intellectual humility, and encourage collaborative reflection create conditions in which knowledge can flow freely and be integrated effectively (Aarons et al., 2020). Effective leaders also help teams maintain alignment through shared visioning, goal-setting, and periodic recalibration.

### 7.3 Integrative strategies and the future of sustainable team science

Sustaining team science is ultimately a matter of integrating structural, relational, cognitive, and cultural elements into a coherent collaborative ecosystem. Institutions can design structures that support team science, but it is the alignment of these structures with supportive social and knowledge-integration processes that determines long-term success.

Sustained team science is therefore characterized by (Cilliers, 2013; Lotrecchiano and Misra, 2020):

- **Institutional supports** that incentivize collaboration.
- **Inclusive relational climates** that promote trust, safety, and mutual respect.
- **Knowledge practices** that enable transparency, integration, and adaptation.
- **Leadership strategies** that distribute power and cultivate learning.
- **Funding and resource structures** that match the non-linear trajectory of interdisciplinary work.

Future research and practice must focus on developing models that account for the complexity and non-linearity of collaborative ecosystems. As scientific challenges grow more interconnected—spanning environmental, technological, social, and health domains—the need for sustainable team science will only intensify. Long-term, resilient, and adaptive team-based research represents not only a methodological preference but an essential infrastructure for addressing twenty-first-century problems. Table 6 provides a Macro-Exo-Meso-Team (MEST) summary with recommendation for implementation of sustaining norms necessary for organizational readiness.

**Table 6:** Summary and recommendations for sustaining team science.

**MEST level**	**Primary focus**	**Key challenges identified in text**	**Sustaining strategies**	**Illustrative mechanisms/examples**
Macro (field, policy, funding environment)	National and disciplinary norms, funding paradigms, and reward systems	•Linear funding models misaligned with non-linear collaboration •Academic norms privileging individual achievement •Undervaluation of interdisciplinary and “invisible” work	•Advocate for flexible, multi-stage, and infrastructure-level funding •Normalize interdisciplinary outputs in evaluation standards •Promote field-wide recognition of team-based expertise	•CTSA and center-based funding models •Federal and foundation calls emphasizing systems science •Revised promotion and tenure guidelines
Exo (institutional/ organizational)	University, research center, or system-level structures	•Misalignment between collaboration demands and institutional policies •PI-centric leadership models •Fragmented infrastructure and governance	•Design coordinated infrastructures for team science •Implement shared or distributed leadership models •Align rewards, evaluation, and resourcing with collaboration	•Shared governance structures •Institutional bridging funds •Recognition of coordination, facilitation, and data stewardship roles
Meso (program, network, or team-of-teams)	Interdisciplinary programs, centers, and collaborative networks	•Cross-disciplinary misunderstandings •Knowledge loss and siloing •Difficulty sustaining innovation over time	•Establish intentional relational and knowledge-integration processes •Use boundary objects and shared vocabularies •Balance stability with adaptability through complex adaptive systems approaches	•Team charters and communication protocols •Knowledge brokers or integrators •Shared digital repositories and dashboards
Team (micro/relational level)	Day-to-day collaboration, relationships, and team climate	•Low psychological safety •Conflict arising from disciplinary differences •Inequitable participation and attrition	•Cultivate trust, respect, and psychological safety •Provide conflict-resolution and communication training •Embed inclusive and equitable collaboration practices	•Reflective dialogue sessions •Mentorship for interdisciplinary identity development •Inclusive meeting norms and role clarity

## 8 Discussion: systematic challenges and the foundations of success in scientific and professional development

In the contemporary landscape of education and professional training, individual success is often attributed to the perseverance, intelligence, or motivation of the person rather than to the systems designed to support them. This misplaced focus can obscure structural deficiencies that limit growth, innovation, and sustainability in the scientific workforce. When institutions celebrate individual achievement without assessing how well the system cultivates such outcomes, they risk perpetuating inequity and burnout while failing to build scalable success models (Austin and Baldwin, 1991; Bilimoria and Bickley, 2014). True progress depends on the capacity of organizations to design environments where individuals thrive because of robust systems—not in spite of their weaknesses (Senge, 2006).

A central issue is the lack of organizational efficacy and systematic evaluation, which significantly constrains the adaptive capacity of educational and research institutions. Too often, organizations rely on outcome indicators of success—such as graduation rates, publication counts, or grant funding—without assessing the deeper processes that enable such achievements (Lotrecchiano et al., 2025). We often refer to these as the taskwork of science over the team work required to conduct science (Fisher, 2014). This reflects a persistent gap between performance measurement and organizational learning (Argyris and Schön, 1996; Preskill and Boyle, 2008). Institutions frequently lack mechanisms to evaluate how effectively their programs foster collaboration, creativity, inclusion, and readiness for complex, interdisciplinary problem-solving. Without structured evaluation frameworks, organizations operate without meaningful feedback, limiting their ability to learn from experience or to respond to emerging challenges (Lotrecchiano et al., 2025; Shea et al., 2014; Weiner, 2020).

Effective evaluation demands a shift from outcome-centered assessment toward process-oriented inquiry that examines the conditions under which learning and collaboration occur. This includes evaluating mentorship quality, team functioning, and the developmental climate of training environments (Lotrecchiano et al., 2016). In implementation science, such reflective practices align with the concept of organizational readiness for change, which emphasizes both the psychological and structural capacity to adapt and sustain new practices (Shea et al., 2014; Weiner, 2009). Institutions that embed evaluation into their culture of practice are better positioned to enact continuous improvement and resilience in the face of complexity (Senge, 2006). Conversely, when organizations neglect these internal feedback loops, they lose the very mechanisms that support growth, innovation, and collective efficacy—key attributes of learning organizations that thrive within dynamic systems (Argyris, 2004).

### 8.1 Inputs and outputs

Too often, systems operate under the assumption that high-quality outcomes—such as research productivity, innovation, or professional readiness—will naturally arise from existing processes, without critically examining or adequately investing in the quality of the inputs that produce them. This assumption reflects a linear and outdated model of organizational effectiveness that ignores the complex interdependencies within modern scientific and educational systems (Checkland, 1999). Inputs are not merely resources but the conditions that enable sustained performance—mentorship, funding stability, infrastructure quality, access to collaboration networks, and psychological safety (Feldman et al., 2010). Each of these elements shapes the developmental trajectory of scientists and trainees, influencing not only what they achieve but how they engage in learning and discovery (Austin and Baldwin, 1991). The intentional design of these input conditions is critical. Mentorship quality predicts long-term research engagement and career satisfaction (Feldman et al., 2010; Pfund et al., 2016), while inclusive climates and psychological safety foster openness, creativity, and effective team functioning (Lotrecchiano et al., 2016; Edmonston, 1999). Without such investment, organizational systems risk producing inconsistent and inequitable outcomes, where success depends on individual resilience rather than systemic support (Bilimoria and Bickley, 2014). This imbalance reinforces a narrative that attributes achievement to personal drive, obscuring institutional responsibility in creating equitable and enabling environments (Argyris, 2004).

Ultimately, alignment between expectations and resources is essential for coherence between inputs and outputs. When expectations exceed available support, organizations generate dissonance that undermines motivation, trust, and long-term innovation capacity—further widening the gap between individual excellence and collective efficacy (Weiner, 2020; Shea et al., 2014)

### 8.2 Scaffolding and competency development

Part of this misalignment stems from curricula that lack adequate scaffolding to cultivate the competencies necessary for a successful and adaptive scientific workforce (Brasier et al., 2023; Lotrecchiano et al., 2020; Mendell et al., 2024). Many educational and training programs continue to prioritize technical expertise—the mastery of specialized methods, procedures, and content knowledge—while underemphasizing the integrative skills essential for modern science, such as systems thinking, collaboration, communication, and adaptive problem-solving (Fairchild et al., 2018; Hall et al., 2018). This imbalance reflects an outdated educational paradigm in which learning is viewed as the accumulation of discrete knowledge rather than the development of transferable competencies that enable innovation across complex, interdisciplinary environments (Bransford et al., 2000).

Scaffolding, in educational theory, refers to the structured and progressive support that connects theoretical understanding to practical application (Vygotsky, 1978). In scientific training, this may involve mentoring, experiential learning, reflective practice, and interprofessional collaboration exercises that gradually foster autonomy and competence (Bransford et al., 2000; Pfund et al., 2016). When such scaffolds are insufficient, learners may achieve technical proficiency but lack the integrative capacity to apply knowledge creatively across domains or to engage effectively in team-based work. Moreover, failure to scaffold learning that bridges disciplinary and professional boundaries contributes to fragmentation within the scientific enterprise, hindering both collaboration and innovation (Hall et al., 2018).

### 8.3 Systems-based science and adaptive capacity

Systems-based sciences, by contrast, emphasize interdependence, adaptability, and contextual awareness—qualities essential for addressing the complex problems facing contemporary research and society (Checkland, 1999; Rouse, 2008). Unlike traditional reductionist approaches, which focus narrowly on isolated variables or individual performance, systems-based approaches examine how components interact within dynamic networks, whether in biological, organizational, or social contexts (Senge, 2006; Falk-Krzesinski and Klein, 2017). Disciplines such as implementation science, team science, and systems biology exemplify this perspective, recognizing that robust solutions emerge not from individual effort alone but from the coordinated interaction of people, processes, and institutions (Lotrecchiano et al., 2016; Cooke and Hilton, 2015).

These frameworks highlight the importance of adaptive capacity—how teams and organizations must continuously learn, iterate, and respond to evolving challenges (Weiner, 2020). In team science, success depends on aligning expertise, incentives, and communication across disciplinary and cultural boundaries (Klein, 1996; Falk-Krzesinski and Klein, 2017; Lotrecchiano et al., 2016). Systems-based approaches also underscore contextual awareness, acknowledging that outcomes are shaped by institutional structures, cultural norms, and resource constraints (Argyris and Schön, 1996). By designing processes that leverage these factors, systems-based sciences amplify human potential, fostering collaboration, innovation, and sustainable success (Senge, 2006). In contrast to models that privilege individual achievement in isolation, systems-based approaches demonstrate that effective science is inherently collective, iterative, and adaptive (Falk-Krzesinski and Klein, 2017; Rouse, 2008). Rather than relying on the performance of a single “star” performer, these approaches recognize that meaningful outcomes emerge from the coordinated efforts of teams operating in feedback-rich environments. By emphasizing shared decision-making, iterative problem-solving, and cross-functional integration, systems-based frameworks provide a roadmap for structuring curricula and institutions to transform raw talent into coordinated, impactful, and resilient outcomes (Lotrecchiano et al., 2016; Hall et al., 2018). Such approaches make explicit the mechanisms by which collaboration, mentorship, and institutional support amplify individual contributions, turning isolated skills into sustainable collective capability (Senge, 2006; Edmonston, 1999).

### 8.4 Cultural and cognitive integration

Integration is complicated by cultural divides across disciplines, institutions, and generations. Differences in communication, perceptions of authority, and problem-solving approaches can undermine trust and reduce team efficacy (Edmonston, 1999; Hall et al., 2018). Bridging these divides requires deliberately cultivating adaptive and creative thinkers—individuals who are reflective, innovative, and comfortable reimagining established paradigms (Runco, 2004; Mumford et al., 2010). These thinkers thrive in environments that reward exploration and collaboration rather than conformity or output-driven metrics.

By intentionally designing systems that integrate collective processes, account for cultural variation, and nurture adaptive cognition, organizations can build resilient scientific ecosystems that maximize both individual potential and collective capability. Such ecosystems ensure that innovation is achieved and sustained across teams, disciplines, and institutional contexts (Lotrecchiano et al., 2025; Senge, 2006).

## 9 Conclusion

Readiness for organizational change arises from a constellation of interdependent factors rather than unfolding through a simple, linear progression. Organizational scholars increasingly emphasize that readiness reflects a dynamic state shaped by shifting beliefs, contextual demands, structural enablers, and the evolving relationships among them (Weiner, 2009). From a complexity perspective, readiness is best understood as an emergent property of the system—something that arises through interaction, feedback, and adaptation rather than through top-down planning alone (Uhl-Bien and Marion, 2009). This view challenges conventional models of change and underscores the need for frameworks that appreciate non-linearity, uncertainty, and the interdependencies characteristic of complex organizational environments (Cilliers, 2013).

Equally important is the development of new vocabularies that enable organizations to name, interpret, and negotiate the cultural dimensions of change. While policies and structures may initiate shifts in formal processes, they rarely yield lasting transformation unless accompanied by new shared meanings, narratives, and linguistic tools (Schein, 2010). Complexity theorists similarly argue that language and meaning systems shape how individuals sense and respond to emerging patterns in the environment. When organizations adopt vocabularies that foreground adaptability, relational learning, and collective intelligence, they create the interpretive capacity needed to support—and sustain—transformative change.

Nowhere is this linguistic and conceptual evolution more critical than in team science. As interdisciplinary, cross-sector, and community-engaged collaborations become the norm, effective teamwork relies on a shared lexicon that can bridge diverse epistemologies, methods, and professional cultures. Team science scholarship highlights that robust collaboration depends on communication systems that support integration, coordination, and mutual understanding among varied contributors (National Academies of Sciences Engineering and Medicine, 2025). Complexity-informed research similarly demonstrates that shared language supports the emergence of coherent patterns across distributed networks, enabling groups to navigate uncertainty and co-create new knowledge (Snowden and Boone, 2007). For scientific teams—whether academic, corporate, or citizen-led—such linguistic coherence is essential for achieving synergy and sustaining innovation.

Finally, creating this level of readiness requires the intentional adoption of systems approaches and complexity-based methods. Complexity science frames organizations as adaptive systems in which change emerges from local interactions, non-linear feedback loops, and shifting environmental constraints (Arrow et al., 2000; Mitchell, 2009). Techniques such as systems mapping, adaptive leadership, and network analysis help organizations detect emergent signals, identify leverage points, and design interventions that respond to evolving realities rather than static plans (Uhl-Bien et al., 2007). Without these tools and perspectives, readiness efforts remain fragmented and susceptible to the limitations of linear change models. Ultimately, durable, and scalable organizational transformation depends on fully integrating complexity science and systems thinking into institutional practice, enabling change that is relational, adaptive, and resilient over time.
